# Antimicrobial resistance and molecular characterization of ESBL-producing Enterobacterales from Parirenyatwa Hospital wastewater in Harare

**DOI:** 10.1093/jacamr/dlaf170

**Published:** 2025-10-04

**Authors:** Takudzwa Marembo, Chido Chirenda

**Affiliations:** Africa Centres for Disease Control and Prevention, African Union Commission, Roosevelt Street (Old Airport), Addis Ababa, P.O. Box 3243, Addis Ababa, Ethiopia; Department of Medical Microbiology, Midlands State University Faculty of Medicine, P. Bag 9055, Gweru, Zimbabwe; Medical Microbiology Unit, Faculty of Medicine and Health Sciences, University of Zimbabwe, Harare, Zimbabwe

## Abstract

**Background:**

The hospital environment is a proven hotspot for antimicrobial-resistant bacteria, which may be released through hospital wastewater into the environment and municipal wastewater. The aim of this study was to monitor the occurrence of and perform molecular characterization of MDR ESBL Enterobacterales isolated from Parirenyatwa Hospital wastewater, Harare, Zimbabwe.

**Methods:**

This was a cross-sectional study. Enterobacterales from sixty-four 500 mL samples of hospital wastewater from three drainage sites of Parirenyatwa Hospital were isolated. A modified double disc synergy test was used to confirm ESBL Enterobacterales before genotyping with multiplex PCR.

**Results:**

The majority of isolates came from the main hospital drainage site. All the isolated Enterobacterales showed MDR. Of the 33 Enterobacterales isolated from hospital wastewater, 8 (24%) were ESBL-producing: 5/8 (63%) *Escherichia coli*, 2/8 (25%) *Klebsiella pneumoniae*, and 1/8 (12%) *Citrobacter freundii*. The multiple antibiotic resistance index (MARI) obtained from the ESBL-producing Enterobacterales isolates ranged from 0.5 to 0.75. Seven (87.5%) isolates harboured the *bla*_CTX-M_ gene and five (62.5%) isolates had the *bla*_TEM_ gene, with four (50%) isolates containing both genes. Three isolates contained the *bla*_CTX-M_ gene only and one contained only *bla*_TEM_. The *bla*_SHV_ gene was not detected.

**Conclusions:**

MDR ESBL-producing Enterobacterales were identified from Parirenyatwa Hospital wastewater. The MARI greater than 0.2 indicated that these isolates were from a high-risk source of contamination.

## Introduction

The hospital environment is a potential hotspot for antimicrobial-resistant (AMR) bacteria, which may be released through hospital wastewater into the environment and municipal wastewater.^1^ Hospital wastewater that contains AMR bacteria and genetic determinants of resistance may contribute to the emergence, dissemination and persistence of AMR bacteria in municipal wastewaters.^2^ This wastewater when mixed with municipal wastewater can be introduced into the environment and community. Among the AMR bacteria of public health concern are ESBL-producing Gram-negative bacteria, including Enterobacterales, due to their MDR,^3^ difficulty in treating them,^4^ and their ability to transfer genes encoding antibiotic resistance to antimicrobial-susceptible bacteria through mobile genetic elements such as plasmids, transposons and integrons.^5^

In Harare, Zimbabwe, hospital wastewater is pumped together with municipal wastewater into the Mukuvisi River, a tributary of Lake Chivero.^6^ This in turn has the potential of introducing AMR bacteria contained in hospital wastewater into these two rivers; one study has already isolated AMR bacteria from these water bodies.^7^ This is significant considering that domestic water for Harare is pumped from Lake Chivero, with reports of undertreatment of the water.^8^ Additionally, residents from Harare and surrounding communities conduct recreational activities and fishing in and around Lake Chivero. All these factors have the potential of introducing ESBL-producers and AMR bacteria into the community, which would lead to community-acquired ESBL and AMR bacterial infections whose origin would be linked to the hospital environment. The aim of this study was to monitor the occurrence of MDR ESBL-producing bacteria and perform molecular characterization of the ESBL genes from Enterobacterales isolated from the Parirenyatwa Hospital wastewater in Harare.

## Methods

### Study design

This study was a prospective cross-sectional study of ESBL-producing Enterobacterales isolated from wastewater from the Parirenyatwa group of hospital wards between May 2024 and September 2024.

### Ethical considerations

Ethical approval was obtained from the Joint Parirenyatwa Hospital and College of Health Sciences Research Ethics Committee and Medical Research Council of Zimbabwe (MRCZ/B/2378).

### Sample collection

A total of 32 L of wastewater was collected in sixty-four 500 mL sterile sample containers over a period of 4 months. Sample collection points were waste drainage sites leading from Parirenyatwa Hospital maternity section (Mbuya Nehanda), eye treatment section (Sekuru Kaguvi) and annex for psychiatric patients and several specialist paediatric wards. All the wastewater from the Parirenyatwa Hospital group converges at three drainage outlets, and it is from these three drainage sites that hospital wastewater was collected. The samples were transported on ice to the laboratory and analysed within 24 h of collection.

### Bacterial isolation, identification and antimicrobial susceptibility testing

Hospital wastewater (500 mL) was filtered through nitrocellulose filter paper. The filter paper was then placed on MacConkey agar with bile salts and crystal violet to inhibit growth of Gram-positive bacteria. After 24 h of aerobic incubation at 37°C, the plates were examined for growth, colony morphology and lactose fermentation. All the bacterial colonies obtained from primary culture were Gram stained. All the Gram-negative, rod-shaped, lactose-fermenting colonies were sub-cultured on fresh MacConkey agar to obtain pure colonies. Non–lactose-fermenting, Gram-negative rods and coccobacilli were first tested for oxidase before sub-culturing on fresh MacConkey agar. Oxidase-negative, non–lactose-fermenting, Gram-negative bacilli and coccobacilli were sub-cultured. Additionally, oxidase-negative coccobacilli were further cultured on Kligler iron agar to rule out *Acinetobacter* species. Gram-negative rods from sub-cultured plates were retested for oxidase using oxidase strips. Gram-negative/oxidase-negative/glucose-fermenting bacterial colonies (presumptive Enterobacterales) were identified to species level using API (Analytical Profile Index kit; Cypress Diagnostics, Hulshout, Belgium). *Escherichia coli* ATCC 25922 was used as a control strain. All confirmed Enterobacterales were tested for antimicrobial susceptibility using the Kirby–Bauer disc diffusion method as per CLSI guidelines using ampicillin (10 μg), ceftriaxone (30 μg), nalidixic acid (30 μg), amoxicillin/clavulanic acid (20/10 μg), cefepime (30 μg), ciprofloxacin (5 μg), chloramphenicol (30 μg), gentamicin (30 μg), ceftazidime (30 μg), ertapenem (10 μg), nitrofurantoin (100 μg) and cefotaxime (30 μg).^9^ Isolates with inhibition zone diameters of ≤27 mm and ≤22 mm for ceftriaxone (30 μg) and ceftazidime (30 μg), respectively, were categorized as presumptive ESBL-producing isolates and taken for phenotypic confirmation.

### Phenotypic detection of ESBLs

Confirmation of ESBL production was done using the modified double disc synergy test (MDDST) using amoxicillin/clavulanate (20/10 μg) and five cephalosporins, both third- and fourth-generation.^9,10^ Third-generation cephalosporins used included ceftriaxone (30 μg), cefpodoxime (10 μg), ceftazidime (30 μg) and cefotaxime (30 μg). Cefepime (30 μg), a fourth-generation cephalosporin, was used to cater for AmpC producers. *E. coli* ATCC 25922 and *Klebsiella pneumoniae* ATCC 700603 were used as negative and positive controls for ESBL production, respectively. All pure isolates positive for MDDST were then stored in glycerol stocks awaiting ESBL genotyping.

### DNA extraction and amplification by multiplex PCR

DNA from bacterial colonies cultured overnight on blood agar was extracted using the boiling method.^11^ Multiplex PCR to detect *bla*_CTX-M_, *bla*_TEM_ and *bla*_SHV_ genes was done as described by Khanfar.^12^ The PCR reaction had a final volume of 25 µL: 10 µM (1 µL) of each forward and reverse primer (Table 1), 12.5 µL of One Taq Quick-Load 2X Master Mix (Inqaba Biotechnical Industries, Gauteng, South Africa), 6.5 µL total bacterial DNA extract and 5 µL molecular grade water. The PCR tubes were immediately transferred to a DNA thermocycler (Eppendorf Master cycler, Hamburg, Germany). The PCR was run under the following conditions: 95°C for 5 min for initial denaturation, followed by 30 amplification cycles of 30 s at 95°C, 30 s at 54°C and 120 s at 72°C. The final extension step was done at 72°C for 10 min. PCR products were analysed by 1.5% gel electrophoresis, and the band sizes were used to identify the ESBL genes using a 1 kb molecular weight marker (New England Biolabs, UK).

**Table 1.** ESBL type-specific primers for multiplex PCR^12^

Primer	Forward	Reverse	Size, bp
*bla*_SHV_	ATGCGTTATATTCGCCTGTG	TGCTTTGTTATTCGGGCCAA	747
*bla*_TEM_	TCGCCGCATACACTATTCTCAGAATGA	ACGCTCACCGGCTCCAGATTTAT	445
*bla*_CTX-M_	ATGTGCAGYACCAGTAARGTKATGGC	TGGGTRAARTARGTSACCAGAAYCAGCGG	593

### Data analysis

Data from the study were entered into Microsoft Excel 2013 (Microsoft Corporation, Redmond, WA, USA) and imported to SPSS Statistics 27.0.1.0 for statistical analysis. The multiple antibiotic resistance index (MARI) of bacterial species was calculated by dividing the number of antibiotics that an isolate is resistant to by the total number of antibiotics used. A MARI >0.2 indicates that the bacteria are from a high-risk source of contamination.

## Results

A total of sixty-four 500 mL containers of wastewater were collected from three different wastewater drainage sites, namely Mbuya Nehanda maternity ward, General Hospital and Sekuru Kaguvi eye treatment clinic, to give a total of 32 L from the sites over a period of 4 months. Growth on MacConkey agar was obtained from all the 64 wastewater samples. A total of 39 Gram-negative bacilli and coccobacilli were sub-cultured on MacConkey agar from the primary plates after exclusion of Gram-positive and oxidase-positive bacteria tested from primary culture plates. Thirty-three (85%) of these were presumptive Enterobacterales after excluding six coccobacilli that tested negative for glucose fermentation. Presumptive Enterobacterales (*n* = 33) isolates were identified to species level using Analytical Profile Index (API) tests.

### Distribution of Enterobacterales per drainage site across Parirenyatwa Hospital

**Figure 1.**
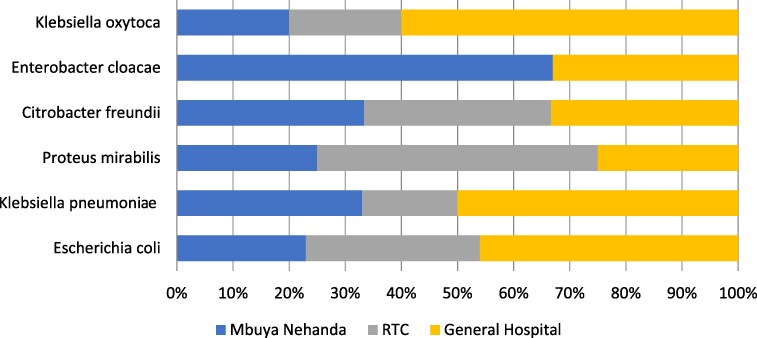
Distribution of Enterobacterales across the three collection sites from Parirenyatwa Group of hospitals. RTC, Radiotherapy Centre.

The predominant bacterial species isolated in this study was *E. coli* (*n* = 13 isolates; 40%), followed by *K. pneumoniae* (*n* = 6; 18%), *Klebsiella oxytcoa* (*n* = 5; 15%), *Proteus mirabilis* (*n* = 4; 12%), *Enterobacter cloacae* (*n* = 3; 9%) and *Citrobacter freundii* (*n* = 2; 6%). Most (*n* = 15; 46%) isolates were collected from the General Hospital drainage site, i.e. 46% (*n* = 6) of *E. coli*, 60% (*n* = 3) of *K. oxytoca* and 50% (*n* = 3) of *K. pneumoniae*. Two (67%) *E. cloacae* isolates were collected from hospital wastewater obtained from the Mbuya Nehanda collection site, with two (50%) *P. mirabilis* isolated from the radiotherapy centre collection site as shown in Figure 1.

### Antimicrobial susceptibility profiles

Antimicrobial susceptibility testing was done on all 33 isolates. All isolates were susceptible to imipenem and resistant to nalidixic acid. Intermediate resistance was observed for Augmentin (amoxicillin/clavulanic acid) by 21% (7/33) of isolates. MDR was observed in all the collected bacterial species.

### Occurrence of ESBL-producing Enterobacterales isolates

Eight (24%, 8/33) isolates were ESBL-producers, including: five *E. coli*, two *K. pneumoniae* and one *C. freundii* isolate.

### Multiple antibiotic resistance phenotypes and MARIs of ESBL-producing isolates’

The ESBL-producing isolates were considered to be MDR when they showed resistance against three or more different classes of antibiotics. Table 2 shows the different multiple antibiotic resistance phenotypes (MARPs) and MARIs from the eight ESBL-producing isolates. All eight isolates exhibited phenotypic resistance against a minimum of 6 out of the 12 tested antibiotics. The MARI of these isolates ranged from 0.5 to 0.75.

**Table 2.** Antimicrobial resistance profiles of ESBL-producing Enterobacterales

Bacteria	Antibiogram	Number of antibiotics to which resistance was shown	Number of phenotypes observed	MARI
*E. coli*	SXT,CAZ,CTX,NAL,CIP,FEP,NIT,CRO,AMP	9	1	0.75
*E. coli*	SXT,CTX,CAZ,NAL,CIP,FEP	6	1	0.50
*E. coli*	STX,CTX,CAZ,NALL,CIP,FEP,CRO,AMP	8	2	0.66
*E. coli*	SXT,CAZ,FEP,GEN,AMP,CTX	6	1	0.50
*E. coli*	CAZ,CTX,SXT,FEP,AMP,CRO	6	1	0.50
*K. pneumoniae*	AMP,CRO,FEP,CAZ,NAL,CTX	6	1	0.50
*K. pneumoniae*	AMP,NIT,CFP,CRO,NAL,GEN	6	1	0.50
*C. freundii*	AMP,CTX,CAZ,FEP,NAL,GEN,SXT	7	1	0.58

AMP, ampicillin; CAZ, ceftazidime; CIP, ciprofloxacin; CRO, ceftriaxone; CTX, cefotaxime; FEP, cefepime; GEN, gentamicin; NAL, nalidixic acid; NIT, nitrofurantoin; SXT, trimethoprim/sulfamethoxazole (cotrimoxazole).

### ESBL genotypes

**Figure 2.**
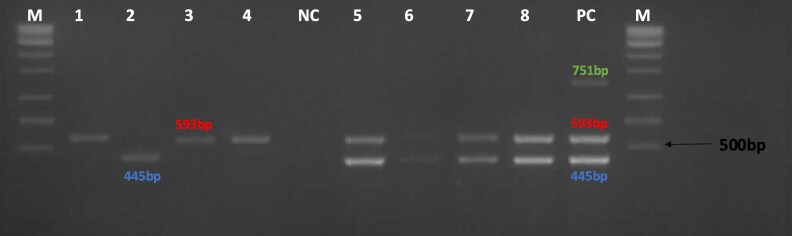
Multiplex PCR band patterns for ESBL genotypes. M, 1 kb plus DNA ladder; NC, negative control (nuclease-free water); PC, positive control (CTX-M, TEM and SHV); lanes 1–8 = samples.

The eight (100%) ESBL-producing isolates were positive for ESBL genes with PCR. Seven and five isolates harboured *bla*_CTX-M_ and *bla*_TEM_ genes, respectively. In addition, four contained both *bla*_CTX-M_ and *bla*_TEM_ genes. Three isolates harboured *bla*_CTX-M_ only whereas one harboured *bla*_TEM_ only. The *bla*_SHV_ gene was not detected in any isolate. Figure 2 depicts gel electrophoresis with the amplified genes.

## Discussion

This study isolated Enterobacterales from hospital wastewater from the Parirenyatwa group of hospitals. *E. coli* was the most frequent, followed by *K. pneumoniae*, *P. mirabilis*, *C. freundii*, *E. cloacae* and *K. oxytoca.* In a study carried out in South Africa, the bacteria isolated from hospital wastewater were *K. oxytoca* (36%), *K. pneumoniae* (29%), *E, coli* (10%), *C. freundii* (10%) and *Enterobacter asburiae* (6%).^13^ This contrasts with the findings in our study in which the predominant species isolated was *E. coli* (40%). In a related study done in Taiwan, *Citrobacter* species (10.3%), *Klebsiella* species (11.3%), *Enterobacter* species (19.8%) and *E. coli* (32.9%) were the predominant bacterial isolates from hospital wastewater.^14^ The order of prevalence of the other isolated species may be different but the results of all these studies give an insight into the consortium of Enterobacterales members that are frequently isolated from hospital wastewater. The findings of this study also compare well with those mentioned above regarding the species that are commonly identified in hospital effluents from different countries.

In a study from Nigeria,^15^ a prevalence of 15.5% of ESBL-producing bacteria from 32 L of hospital wastewater was reported, which is consistent with the 24% prevalence of our study. A total of eight ESBL-producing organisms were identified in our study, namely *E. coli* (*n* = 5; 63%), *K. pneumoniae* (*n* = 2; 25%) and *C. freundi* (*n* = 1; 12%). A study by Chandran *et al.*^16^ reported a prevalence of 96% for ESBL-producing *E. coli* in hospital wastewater, much higher than that in our study. Other studies have reported prevalences of 25%, 25% and 37%.^17–19^ All these were resistant against three or more classes of drugs and were thus classified as MDR. This may be due to the ability of the bacteria to survive for long periods and multiply in wastewater leading to selection and emergence of ESBL-producing *E. coli*.^20^

The MARI for the ESBL-producing Enterobacterales ranged from 0.5 to 0.75. A MARI >0.2 indicates that the bacteria are from a high-risk source of contamination.^21^ High MARI values suggest that the bacterial isolates were retrieved from an environment that exerts a high level of selective pressure for antibiotic resistance. The findings of our study are similar to those of a Polish study of hospital effluents in which 48.4% of the Enterobacterales isolates displayed resistance against the majority of the antibiotics used.^22^

Molecular characterization of the ESBL isolates showed a predominance of *bla*_CTX-M_ followed by *bla*_TEM_. The *bla*_SHV_ gene was not detected in our study. These findings are consistent with literature in which CTX-M β-lactamase has been reported as the predominant enzyme amongst the ESBLs, followed by TEM β-lactamase.^23,24^ In India, a higher prevalence of *bla*_CTX-M_ compared with *bla*_TEM_ in hospital wastewater was reported.^16^ ESBL genes were found in *E. coli* (5), *K. pneumoniae* (2) and *C. freundii* (1) making a total of eight ESBLs. These findings in which ESBL genes were more frequent in *E. coli* agree with those reported in a study in Nigeria in which five ESBLs were reported: three *E. coli*, one *K. pneumoniae* and two *P. aeruginosa*.^15^ The predominance of *bla*_CTX-M_ in *E. coli* reported in our current study has also been reported worldwide.^25^ A study done in Thailand reported a prevalence of 99.6% and 99.2% of the *bla*_CTX-M_ gene in *E. coli* and *K. pneumoniae*, respectively.^26^ In the same study, up to 77.0% and 71.7% of the ESBL-producing *E. coli* and *K. pneumoniae*, respectively, carried the *bla*_TEM_ gene.^26^ Our study did not detect the *bla*_SHV_ gene, a finding that contrasts with that obtained in Thailand, in which *bla*_SHV_ was detected in *E. coli* (3.8%) and *K. pneumoniae* (87.4%).^26^ In as much as SHV-1 is chromosomally encoded and intrinsic to *K. pneumoniae*, all the *K. pneumoniae* isolates from this study were negative for *bla*_SHV_. The results of our study align with findings from other studies that demonstrated the abundance of the *bla*_CTX-M_ gene in ESBL producers isolated from hospital effluents.^22,27^ Four of the isolates in the study harboured both the *bla*_CTX-M_ and *bla*_TEM_ genes. These findings are in line with a study done in Brazil, in which the majority of the isolates showed an association between *bla*_TEM_ and *bla*_CTX-M_.^28^ Previous studies have shown that this type of association is responsible for high-level β-lactamase resistance phenotypes.^26^ In 2011, in a study done in India, Shahid *et al.*^29^ also reported the coexistence of *bla*_TEM_ and *bla*_CTX-M_. However, the ESBL genes from this study were not sequenced. Sequencing is essential in determining the type of ESBL genes from these isolates, especially for differentiating whether the *bla*_TEM_ genes identified are ESBL variants or *bla*_TEM-1_, a simple penicillinase. One isolate from this study had the *bla*_TEM_ gene alone. However, without gene sequencing to identify the type of *bla*_TEM_ gene, ESBL in four isolates that harboured both *bla*_TEM_ and *bla*_CTX-M_ from this study was identified by the presence of *bla*_CTX-M_.

### Conclusions

MDR ESBL-producing Enterobacterales were identified from Parirenyatwa Hospital wastewater. MARI values >0.2 indicated that these isolates were from a high-risk source of contamination. The most predominant ESBL gene was *bla*_CTX-M_. To improve this study, sequencing of the ESBL genes is recommended in order to determine the sub-types of the different ESBL genes.

## References

[1] Davidova-Gerzova L, Lausova J, Sukkar I et al Hospital and community wastewater as a source of multidrug-resistant ESBL-producing *Escherichia coli*. Front Cell Infect Microbiol 2023; 13: 1184081. 10.3389/fcimb.2023.1184081 37256105 PMC10225658

[2] La Rosa MC, Maugeri A, Favara G et al The impact of wastewater on antimicrobial resistance: a scoping review of transmission pathways and contributing factors. Antibiotics 2025; 14: 131. 10.3390/antibiotics14020131 40001375 PMC11851908

[3] Ibrahim DR, Dodd CER, Stekel DJ et al Multidrug-resistant ESBL-producing *E. coli* in clinical samples from the UK. Antibiotics 2023; 12: 169. 10.3390/antibiotics12010169 36671370 PMC9854697

[4] Husna A, Rahman MM, Badruzzaman ATM et al Extended-spectrum β-lactamases (ESBL): challenges and opportunities. Biomedicines 2023; 11: 2937. 10.3390/biomedicines11112937 38001938 PMC10669213

[5] Benz F, Huisman JS, Bakkeren E et al Plasmid- and strain-specific factors drive variation in ESBL-plasmid spread in vitro and in vivo. ISME J 2021; 15: 862–78. 10.1038/s41396-020-00819-4 33149210 PMC8026971

[6] Nhapi I, Siebel MA, Gijzen HJ. The impact of urbanisation on the water quality of Lake Chivero, Zimbabwe. Water Environ J 2004; 18: 44–9. 10.1111/j.1747-6593.2004.tb00492.x

[7] Takawira H, Mbanga J. Occurrence of multidrug-resistant *Escherichia coli* and antibiotic resistance genes in a wastewater treatment plant and its associated river water in Harare, Zimbabwe. Water SA 2023; 49: 396–403. 10.17159/wsa/2023.v49.i4.4036

[8] Nhapi I . The water situation in Harare, Zimbabwe: a policy and management problem. Water Policy 2009; 11: 221–35. 10.2166/wp.2009.018

[9] CLSI . Performance Standards for Antimicrobial Susceptibility Testing—Thirty-Second Edition: M100. 2023.

[10] Iqbal R, Ikram N, Shoaib M et al Phenotypic confirmatory disc diffusion test (PCDDT), double disc synergy test (DDST), E-test OS diagnostic tool for detection of extended spectrum beta lactamase (ESΒL)-producing uropathogens. J Appl Biotechnol Bioeng 2017; 3: 344–9. 10.15406/jabb.2017.03.00068

[11] Omar BA, Atif HA, Mogahid ME. Comparison of three DNA extraction methods for polymerase chain reaction (PCR) analysis of bacterial genomic DNA. Afr J Microbiol Res 2014; 8: 598–602. 10.5897/AJMR2013.6459

[12] Khanfar H . Molecular epidemiological study of extended spectrum beta-lactamase (ESBL) producing bacteria from a hospital within Saudi Arabia. Doctoral thesis. University of Portsmouth, 2017.

[13] Fadare FT, Okoh AI. Distribution and molecular characterization of ESBL, pAmpC β-lactamases, and non-β-lactam encoding genes in Enterobacteriaceae isolated from hospital wastewater in Eastern Cape Province, South Africa. PLoS One 2021; 16: e0254753. 10.1371/journal.pone.0254753 34288945 PMC8294522

[14] Pärnänen KMM, Narciso-da-Rocha C, Kneis D et al Antibiotic resistance in European wastewater treatment plants mirrors the pattern of clinical antibiotic resistance prevalence. Sci Adv 2019; 5: eaau9124. 10.1126/sciadv.aau9124 30944853 PMC6436925

[15] Egbule OS . Detection and transfer of extended spectrum beta lactamase enzymes from untreated hospital waste water. Adv Microbiol 2016; 6: 512–20. 10.4236/aim.2016.67051

[16] Chandran SP, Diwan V, Tamhankar AJ et al Detection of carbapenem resistance genes and cephalosporin, and quinolone resistance genes along with oqxAB gene in *Escherichia coli* in hospital wastewater: a matter of concern. J Appl Microbiol 2014; 117: 984–95. 10.1111/jam.12591 24975198

[17] Duong HA, Pham NH, Nguyen HT et al Occurrence, fate and antibiotic resistance of fluoroquinolone antibacterials in hospital wastewaters in Hanoi, Vietnam. Chemosphere 2008; 72: 968–73. 10.1016/j.chemosphere.2008.03.009 18485444

[18] Abdulhaq A, Basode VK. Prevalence of extended-spectrum β-lactamase-producing bacteria in hospital and community sewage in Saudi Arabia. Am J Infect Control 2015; 43: 1139–41. 10.1016/j.ajic.2015.06.002 26190382

[19] Diwan V, Chandran SP, Tamhankar AJ et al Identification of extended-spectrum β-lactamase and quinolone resistance genes in *Escherichia coli* isolated from hospital wastewater from central India. J Antimicrob Chemother 2012; 67: 857–9. 10.1093/jac/dkr564 22267239

[20] Zaatout N, Bouras S, Slimani N. Prevalence of extended-spectrum β-lactamase (ESBL)-producing Enterobacteriaceae in wastewater: a systematic review and meta-analysis. J Water Health 2021; 19: 705–23. 10.2166/wh.2021.112 34665765

[21] Osundiya OO, Oladele RO, Oduyebo OO. Multiple antibiotic resistance (MAR) indices of *Pseudomonas* and *Klebsiella* species isolates in Lagos University Teaching Hospital. Afr J Clin Exp Microbiol 2013; 14: 164–8. 10.4314/ajcem.v14i3.8

[22] Korzeniewska E, Harnisz M. Beta-lactamase-producing Enterobacteriaceae in hospital effluents. J Environ Manage 2013; 123: 1–7. 10.1016/j.jenvman.2013.03.024 23563146

[23] Ahlstrom CA, Bonnedahl J, Woksepp H et al Acquisition and dissemination of cephalosporin-resistant *E. coli* in migratory birds sampled at an Alaska landfill as inferred through genomic analysis. Sci Rep 2018; 8: 7361. 10.1038/s41598-018-25474-w 29743625 PMC5943298

[24] Tham J . Extended-spectrum beta-lactamase-producing Enterobacteriaceae: epidemiology, risk factors, and duration of carriage. Doctoral thesis. Department of Clinical Sciences, Lund University, 2012.

[25] Drieux L, Haenn S, Moulin L et al Quantitative evaluation of extended-spectrum β-lactamase-producing *Escherichia coli* strains in the wastewater of a French teaching hospital and relation to patient strain. Antimicrob Resist Infect Control 2016; 5: 9. 10.1186/s13756-016-0108-5 27030806 PMC4812619

[26] Kiratisin P, Apisarnthanarak A, Laesripa C et al Molecular characterization and epidemiology of extended-spectrum- β-lactamase-producing *Escherichia coli* and *Klebsiella pneumoniae* isolates causing health care-associated infection in Thailand, where the CTX-M family is endemic. Antimicrob Agents Chemother 2008; 52: 2818–24. 10.1128/AAC.00171-08 18505851 PMC2493136

[27] Wang Q, Wang P, Yang Q. Occurrence and diversity of antibiotic resistance in untreated hospital wastewater. Sci Total Environ 2018; 621: 990–9. 10.1016/j.scitotenv.2017.10.128 29054666

[28] Chagas TP, Seki LM, Cury JC et al Multiresistance, beta-lactamase-encoding genes and bacterial diversity in hospital wastewater in Rio de Janeiro, Brazil. J Appl Microbiol 2011; 111: 572–81. 10.1111/j.1365-2672.2011.05072.x 21672095

[29] Shahid M, Singh A, Sobia F et al *bla*_CTX-M_, *bla*_TEM_, and *bla*_SHV_ in Enterobacteriaceae from North-Indian tertiary hospital: high occurrence of combination genes. Asian Pac J Trop Med 2011; 4: 101–5. 10.1016/S1995-7645(11)60046-1 21771430

